# Protocol to determine oligomerization states of proteins in living cells using photon counting histogram analysis

**DOI:** 10.1016/j.xpro.2026.104753

**Published:** 2026-08-05

**Authors:** Zixiao Li, Tyler Camp, Yushan Li, Kai Zhang

**Affiliations:** 1 Department of Biochemistry, University of Illinois Urbana-Champaign, Urbana, IL 61801, USA; 2 STC-QCB Center, University of Illinois Urbana-Champaign, Urbana, IL 61801, USA; 3 Cancer Center at Illinois, University of Illinois Urbana-Champaign, Urbana, IL 61801, USA; 4 Center for Biophysics and Quantitative Biology, University of Illinois Urbana-Champaign, Urbana, IL 61801, USA; 5 Neuroscience Program, University of Illinois Urbana-Champaign, Urbana, IL 61801, USA; 6 Beckman Institute for Advanced Science and Technology, University of Illinois Urbana-Champaign, Urbana, IL 61801, USA

**Keywords:** Biophysics, Cell culture, Single Cell, Microscopy, Protein Biochemistry, Biotechnology and bioengineering

## Abstract

Determining protein oligomerization states in living cells is essential for understanding cell signaling and disease pathology, but it remains technically challenging. Here, we present a protocol for quantifying the oligomeric states of HaloTag-labeled proteins in living mammalian cells using fluorescence fluctuation spectroscopy (FFS) and photon counting histogram (PCH) analysis. Using the photoactivatable protein cryptochrome 2 as a representative model, we describe steps for cell culture, chemical transfection, cell staining, and FFS data acquisition. We then detail procedures for PCH analysis.

For complete information on the generation and use of this protocol, please refer to Camp et al.^1^

## Highlights

• Guidance on calibration of confocal microscopes for quantitative FFS measurements
• Instructions to prepare cells and fluorescently label proteins for FFS
• Steps for acquiring FFS data during optogenetic-light stimulation
• Steps for PCH analysis to extract concentration and molecular brightness

## Abstract (editor highlights)

Publisher’s note: Undertaking any experimental protocol requires adherence to local institutional guidelines for laboratory safety and ethics.

## Graphical abstract

**Figure:**
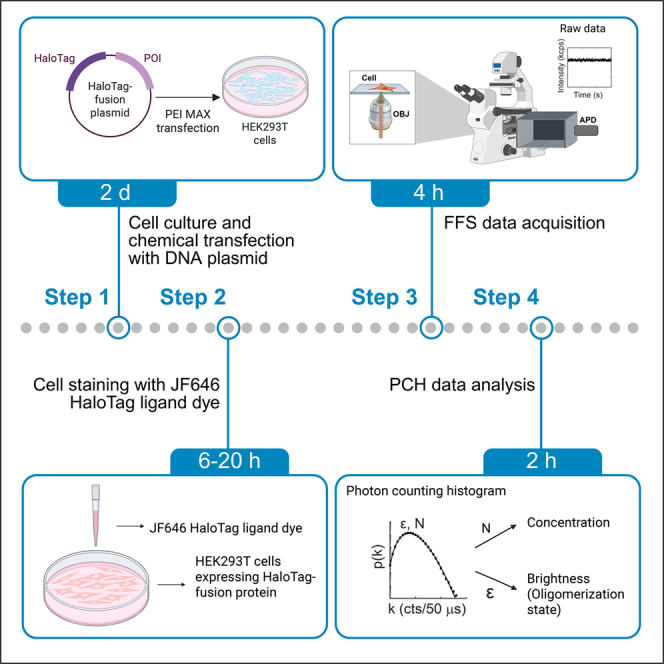

## Before you begin

Protein oligomerization is critical for the formation of biomolecular condensates underlying physiological or pathological conditions.^2^ Although in vitro methods, such as biochemical assays, crystallography, and cryo-electron microscopy (Cryo-EM),^3^ provide insights into the protein assembly state, a quantitative understanding of this dynamic process in living cells is limited. In this protocol, we describe a procedure for quantifying the oligomerization states of HaloTag-labeled proteins using fluorescence fluctuation spectroscopy (FFS)^4^ combined with photon counting histogram (PCH) analysis.^5^^,^^6^^,^^7^ FFS is a fluorescence-based method that records fluctuations in fluorescence intensity as fluorescent molecules diffuse into and out of the tiny observation volume. Conventional imaging techniques measure average fluorescence intensity and cannot differentiate the oligomerization states; fluorescence resonance energy transfer (FRET) detects intra- or inter-molecular nanometer proximity but does not directly give stoichiometry (of the oligomer); super-resolution imaging resolves spatial structure of macromolecular complex but often suffers from time-consuming data acquisition and phototoxicity; biochemical assays such as co-immunoprecipitation lose spatial and living-cell context. FFS/PCH enables extraction of molecular properties, such as diffusion, brightness, and concentration in living cells, using fluorescence fluctuation collected over short acquisition time less than 1 min.^7^ HaloTag^8^ is chosen here because it can be labeled with organic dyes that display improved brightness and photostability compared to commonly used fluorescent proteins (FPs).^1^^,^^9^ However, with appropriate calibration, the procedure also works for FPs. The presented procedures cover the full workflow of molecular cloning, mammalian cell culture and transfection, cell staining, instrument setup, data acquisition, and analysis. We elaborate on the optical path and system calibration. To demonstrate its use, we determine the oligomerization state of *Arabidopsis thaliana* CRY2 Photolyase Homology Region (AtCRY2PHR), a widely used photoactivatable protein domain in optogenetics,^10^^,^^11^^,^^12^ in response to blue light stimulation in living cells.

### Innovation

PCH analysis has previously been used to estimate protein oligomerization states by measuring molecular brightness. This protocol extends the application of PCH to quantify real-time protein assembly dynamics in living cells, enabling dynamic characterization of optogenetic actuators under physiologically relevant conditions. By combining PCH-derived molecular brightness measurements with intracellular concentration determination, this workflow resolves concentration-dependent oligomerization states of optogenetic proteins across a broad expression range. Above a threshold intracellular protein concentration, PCH-resolved oligomerization profiles are consistent with structural models obtained from crystallography and cryo-electron microscopy, providing an orthogonal method for validating protein assembly states directly in living cells. Additional technical innovations of this protocol include a systematic comparison of fluorescent labeling strategies for PCH analysis, including HaloTag labeling with synthetic fluorescent ligands and commonly used fluorescent proteins. We demonstrate that HaloTag labeling provides superior performance for PCH measurements by improving molecular brightness determination and oligomerization-state resolution. Overall, this protocol establishes a quantitative framework for linking optogenetic protein concentration, assembly state, and biological activity in living cells.

### Plasmid construction

**Timing: 1 week**

This step describes the workflow for cloning plasmids that encode the HaloTag monomer, the HaloTag tandem dimer, and HaloTag fusion proteins.

***Note:*** The HaloTag monomer contains only one copy of the HaloTag sequence, whereas the HaloTag tandem dimer contains two copies linked by a flexible linker (GGGSGGGS). These two plasmids are necessary for the calibration step to determine the relative brightness between the HaloTag dimer and monomer. The HaloTag fusion protein plasmid contains the protein of interest (POI) and a HaloTag on either the N-terminus or the C-terminus of the POI. This protocol uses an N-terminal HaloTag fusion. A Ubiquitin C (UBC) promoter is used in all constructs.^13^

**CRITICAL:** In practice, always check the perturbation effect of fusion tags on the POI. Avoid placing HaloTag at positions that could interfere with protein function. Other molecular tags, such as SnapTag, can also be used.

1. Construct the HaloTag monomer plasmid.
  a. Linearize the pUBC vector backbone (refer to pUBC-EGFP plasmid in the key resources table) by BamHI digestion.
    **CRITICAL:** In our experience, the UBC promoter results in protein expression at sub-micromolar levels in HEK293T cells, thereby enabling the collection of high-quality FSS data and PCH analysis.
  b. Amplify the HaloTag insert with overlap extension PCR to generate complementary overhangs. (Refer to HaloTag as the template DNA and 1X-HT.FOR and 1X-HT.REV as the primers in the key resources table).
  c. Ligate the backbone and the insert using the InFusion HD Enzyme.
    ***Note:*** The HaloTag monomer plasmid includes a BamHI site before the Kozak sequence, a BglII site and an EcoRI site after the coding sequence but before the stop codon. The inclusion of BamHI and BglII cut sites positioned this way allows for tandem cloning of multiple copies of the HaloTag sequence using an identical PCR product. (Figure 1A).

**Figure 1:**
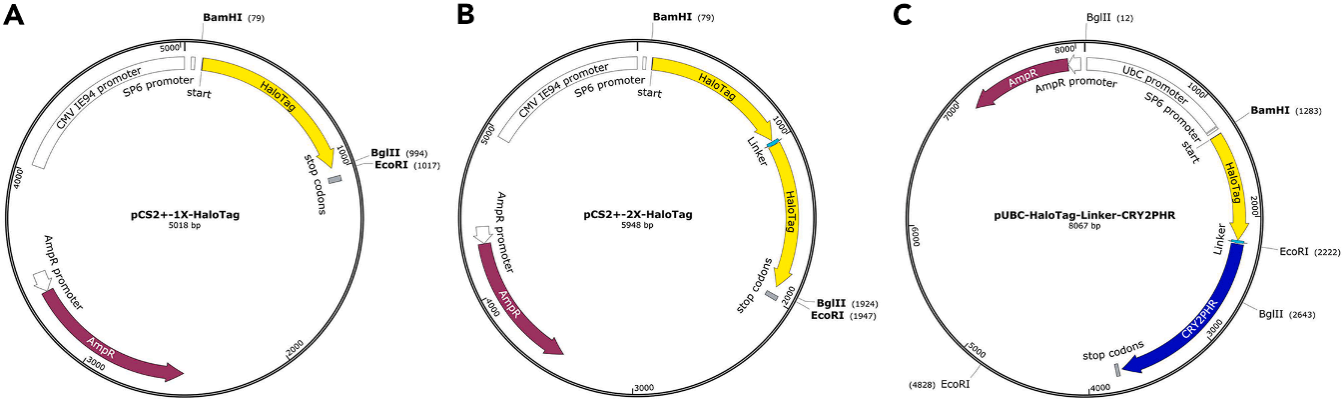
Schematic plasmid maps (A) Schematic drawing of pCS2+-1×-HaloTag plasmid map with restriction enzyme recognition sites. (B) Schematic drawing of pCS2+-2×-HaloTag plasmid map with restriction enzyme recognition sites. (C) Schematic drawing of pUBC-HaloTag-Linker-CRY2PHR plasmid map with restriction enzyme recognition sites.
2. Construct the HaloTag tandem dimer plasmid. (Figure 1B).
  a. Digest the monomer plasmid (pCS2+-1×HaloTag) with BglII/EcoRI to generate the new vector backbone.
  b. Add the flexible glycine/serine linker (GGGSGGGS) between two copies of HaloTag by amplifying the second HaloTag sequence with PCR, with the linker and BamHI restriction site added via primer extension. (Refer to pUBC-1X-HaloTag as the template DNA and HT-Linker.FOR and 1X-HT.REV as the primers in the key resources table).
  c. Digest the PCR product with BamHI/EcoRI.
  d. Remove the phosphates of each plasmid vector with recombinant shrimp alkaline phosphatase (rSAP).
  e. Ligate the new vector backbone and the digested PCR product using T4 DNA ligase.
    ***Note:*** Because BamHI and BglII produce identical 5'-GATC sticky overhangs, the digested vector and PCR products can be ligated to construct head-to-tail repeats. We initially used the high-level expression pCS2+ vector to construct the HaloTag monomer and dimer plasmids. To adjust the protein expression level appropriate for PCH analysis, we then cloned the whole transgene into the low-level expression pUBC vector.
3. Construct the HaloTag-POI fusion plasmid.
  a. Amplify the POI sequence with PCR, with an N-terminal EcoRI site and a KpnI site after the stop codon added via primer extension.
    ***Note:*** We used *Arabidopsis thaliana* cryptochrome 2 (AtCRY2) as the POI in this study. (Refer to mCh-RIPK3-AtCRY2 as the template DNA and Cterm-CRY2.FOR and Cterm-CRY2.REV as the primers) (Figure 1C).
  b. Double-digest the vector plasmid (refer to pUBC-1X-HaloTag in the key resources table) and the PCR insert with EcoRI and KpnI enzymes.
    ***Note:*** The vector plasmid contains the HaloTag sequence followed by the flexible linker/EcoRI site before the stop codon, and a KpnI site after the stop codon.
  c. Remove the phosphates of each vector fragment with rSAP.
  d. Ligate the vector backbone and the digested PCR insert product using T4 DNA ligase.
4. Deliver the ligated plasmid product (amount of DNA depends on competent bacterial strain used) into competent bacteria using standard cloning methods, e.g., heat shock.
  a. After heat shock, incubate cells in LB broth to express antibiotic-resistance genes.
  b. Plate the cell-containing LB broth on a selective agar plate to isolate transformed colonies.
5. Screen for colonies by plasmid extraction, followed by restriction digestion with plasmid-specific enzymes or colony PCR from 6–12 selected colonies.
6. Confirm the plasmid sequence by sequencing before further experiments.

### Coat glass-bottom dishes with poly-L-lysine coating buffer

**Timing: 2 h**

This step describes the procedure to coat glass-bottom tissue-culture dishes or plates with poly-L-lysine (PLL), which provides a positively charged surface to facilitate better cell adherence.

***Note:*** All steps should be carried out in a sterile biosafety cabinet.

7. Make PLL stock solution aliquots.
  a. Dissolve PLL powder in 0.1 M borate buffer (pH 8.5) to make a 10 mg/mL stock solution.
  b. Divide PLL stock solution into aliquots of 0.5 mL and store at −20 °C.
8. Dilute PLL stock in 0.1 M borate buffer (pH 8.5) to make 0.1 mg/mL PLL coating buffer and add to the cell culture dish to cover the bottom and incubate for at least 1 h (up to overnight).
9. Aspirate PLL coating buffer.
10. Wash the dish with autoclaved water 3 times.
11. Air dry the dish in the sterile biosafety cabinet.

## Key resources table

**Table:** 

REAGENT or RESOURCE	SOURCE	IDENTIFIER
**Bacterial and virus strains**		

Stellar Competent Cells	Takara	Cat#636763
DH5α Competent Cells	UIUC Cell Media Facility	N/A

**Biological samples**		

FastDigest BamHI	ThermoFisher Scientific	Cat#FD0054
FastDigest BglII	ThermoFisher Scientific	Cat#FD0083
FastDigest EcoRI	ThermoFisher Scientific	Cat#FD0274
FastDigest KpnI	ThermoFisher Scientific	Cat#FD0524
rCutSmart buffer	New England Biolabs	Cat#B6004S
T4 DNA ligase buffer	New England Biolabs	Cat#B0202S
recombinant shrimp alkaline phosphatase	New England Biolabs	Cat#M0371S
T4 DNA ligase	New England Biolabs	Cat#M0202S
Q5 Site-Directed Mutagenesis Kit	New England Biolabs	Cat#E0554S
InFusion HD Cloning Kit	Takara Bio USA, Inc.	Cat#639650
Atto655, free acid	ATTO-TEC GmbH	Cat#AD 655-21
Atto565, free acid	ATTO-TEC GmbH	Cat#AD 565-21
Janelia Fluor 646 HaloTag, 1 nmol	Promega	Cat#HT1060
Tween 20	Millipore Sigma	Cat#P7949
Dulbecco’s Modified Eagle Medium	Corning	Cat#10-013-CV
Penicillin/streptomycin	Corning	Cat#30-001-CI
Opti-MEM with L-glutamine, no phenol red	Gibco	Cat#11058021
Trypsin + 0.25% EDTA	Gibco	Cat#25200-056
Fetal bovine serum	Avantor	Cat#76419-584
PEI Max	Polysciences	Cat#24765(1)
Glass-bottom 35 mm dishes, no. 1.5 coverslip	Mattek	Cat# P35G-1.5-14-C

**Chemicals, peptides, and recombinant proteins**		

Poly-L-lysine	Millipore Sigma	Cat#P1274
Boric acid	Millipore Sigma	Cat#B6768

**Experimental models: Cell lines**		

HEK293T	ATCC	Cat#CRL-3216

**Oligonucleotides**		

1X-HT.FOR	TCTTTTTGCAGGATCCGCCACCATG GCAGAAATCGGTACTGGC	Build HaloTag monomer plasmid
1X-HT.REV	CGAATCGATGGGATCTTAAGATC TAGTGGTTGGCTCGCC	Build HaloTag monomer plasmid
HT-Linker.FOR	ATGATCGGATCCGGAGGTGGAAGCG GTGGAGGCTCAGCAGAAATCGG TACTGGCTTTCC	Add flexible linker to HaloTag tandem dimer
Nterm-HT-Linker.FOR	GGTGGAGGCTCAGAATTCA AGGCCTCTCGAG	Add flexible linker to HaloTag
Nterm-HT-Linker.REV	GCTTCCACCTCCAGTGGTTGGCTCGCC	Add flexible linker to HaloTag
Cterm-CRY2.FOR	CCATCGATTCGAATTCATGAAGATGG ACAAAAAGACCATCGT	Generate CRY2 sequence with EcoRI site
Cterm-CRY2.REV	GAGAGGCCTTGAATTTCAGCTAGCG GCAGCACCGATCATAAT	Generate CRY2 sequence with EcoRI site

**Recombinant DNA**		

pUBC-EGFP	Addgene	#169746
pUBC-1X-HaloTag	Camp et al.^1^	Addgene # 260112
pUBC-2X-HaloTag	Camp et al.^1^	Addgene # 260113
pUBC-HaloTag-Linker-CRY2PHR	Camp et al.^1^	Addgene # 260114
mCh-RIPK3-AtCRY2	Oh et al.^12^	Addgene # 260127
HaloTag	Gift from Dr. Jason D. Shepherd, University of Utah	N/A
pCS2+-1XHaloTag	Dr. Kai Zhang lab plasmid stock, University of Illinos at Urbana-Champaign	N/A
pCS2+-2X-HaloTag	Dr. Kai Zhang lab plasmid stock, University of Illinos at Urbana-Champaign	N/A

**Software and algorithms**		

VistaVision	ISS Inc. Version (64) 4.2.584	https://iss.com/
Fiji	Schindelin et al.^14^	https://imagej.nih.gov/ij/

**Other**		

Microscope	Olympus	IX73
Scanning mirrors module	ISS Inc.	M612
3-X DAC control card	ISS Inc.	M600
4-Channel Correlation card	ISS Inc.	M230
Single Photon Counting Avalanche Photodiode	Excelitas Technologies	SPCM-AQRH

## Materials and equipment

**Table:** 0.1 M Borate buffer (pH 8.5) for PLL solution preparation

Reagent	Final concentration	Amount
Boric acid	0.1 M	3.09 g
Sodium hydroxide	1 M	N/A
Milli-Q water	N/A	N/A
Total	N/A	500 mL

Adjust the pH to 8.5 using 1 M NaOH. Store at room temperature (23 ± 2 °C).

### Microscope system setup

**Timing: 1 day with optical alignment**

In this section, we describe our microscopy system as a reference for users wishing to implement the protocol to adapt to their own setups. Our system is a standard laser-scanning confocal microscope equipped with single photon counting avalanche photodiodes (APDs). The main requirement for performing PCH in a confocal setting is a detector capable of single-photon counting. These are typically either photomultiplier tubes (PMTs) with low transit-time spread (TTS), which enable the measurement of photon arrival times, or single-photon APDs. Otherwise, standard confocal microscopy components may be used interchangeably, including dichroic filters, galvanometer scanning mirrors, a scan lens, an objective lens, and an inverted microscope stand. Additionally, PCH may be performed using one-photon or two-photon excitation; the only difference is that these modalities result in different excitation profiles owing to the size of the point spread function (PSF) of the techniques; thus, the brightness must be corrected to compare results between these modalities.^7^

**Figure 2:**
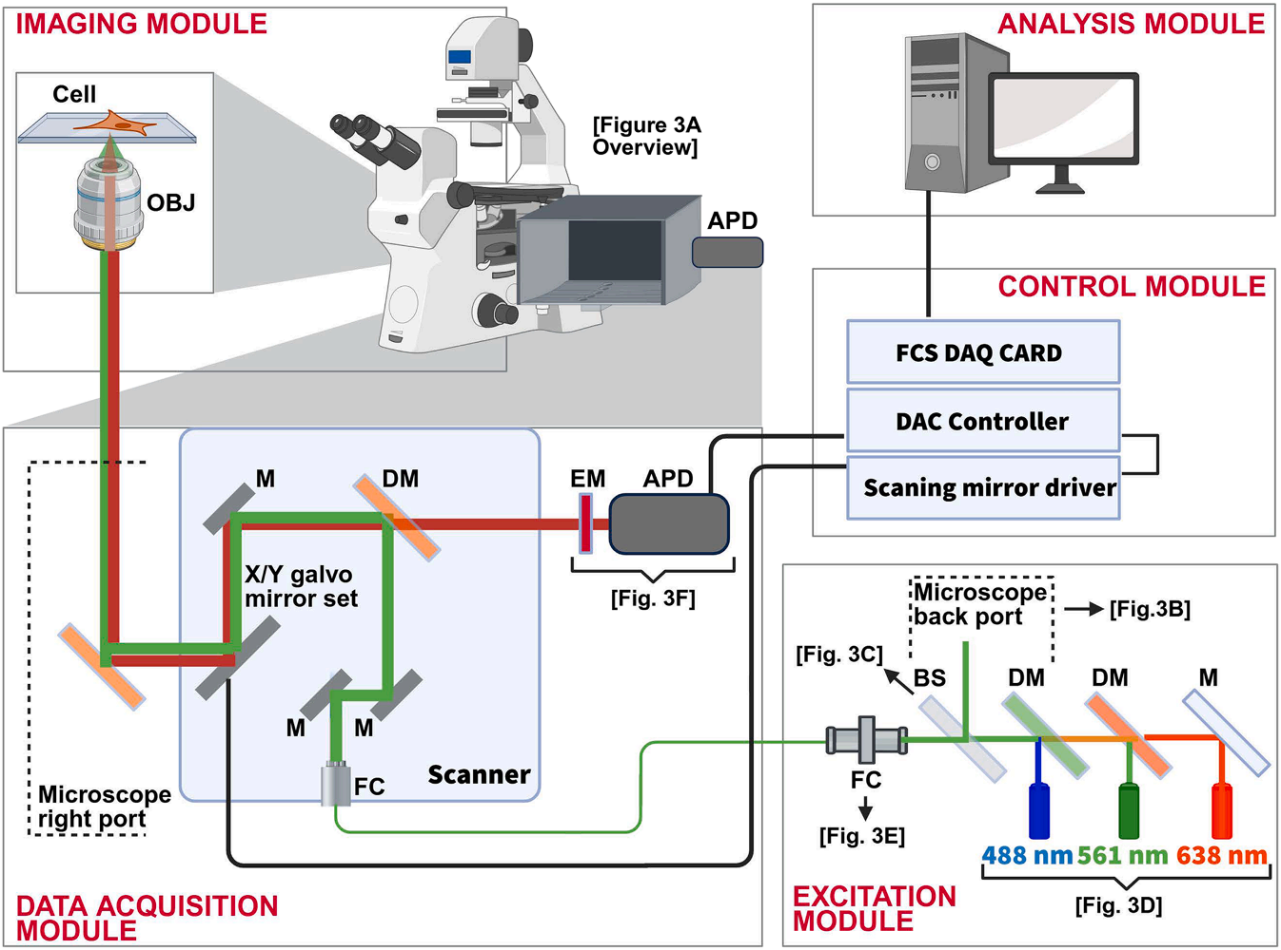
Schematic diagram of the home-built microscope Multiple laser beams are combined and coupled into fiber optics, which direct the excitation beam into the scanner and reflect it into the microscope frame. The excitation laser is focused through the high-NA objective into the cytoplasm of living cells expressing fluorophores. Fluorescence is collected through the same objective and detected by an APD. Data are transmitted into the FCS DAQ card and processed in the computer. Images are acquired by raster scanning two galvo mirrors in the scanner unit, controlled by the scanning mirror driver in the control module.
• An Olympus I×73 microscope is coupled with a galvanometer-driven scanning mirror unit provided by ISS Inc. (Figure 3A).

• The overall design of this home-built microscope is shown as a schematic drawing in Figure 2.

**Figure 3:**
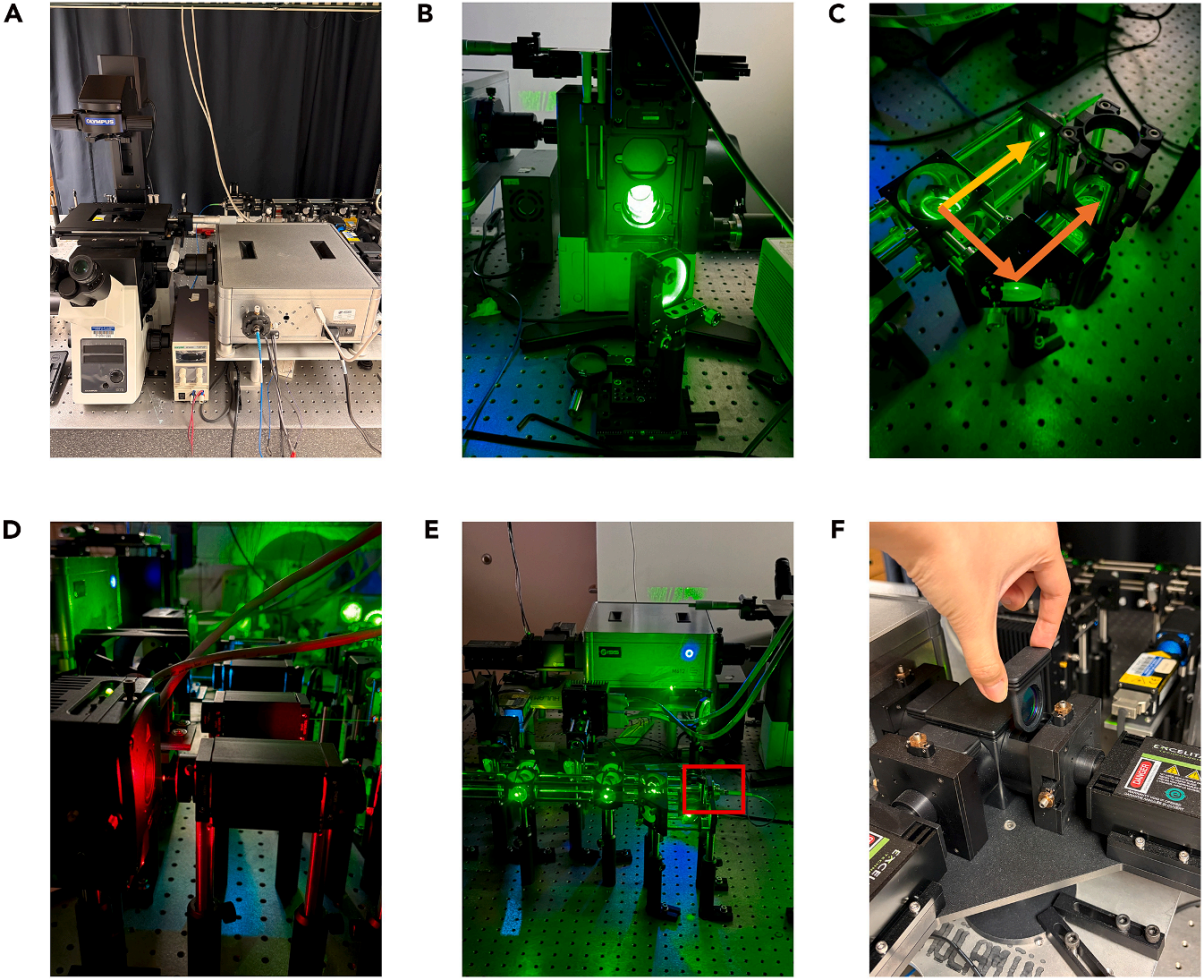
Home-built, multi-color laser excitation microscope with photon counting detector (A) The Olympus IX73 microscope is coupled with an X/Y galvanometer-driven scanning mirror unit. (B) The back port of the microscope, where the excitation laser beam is directed for epifluorescence measurements. (C) The 30/70 beam splitter that splits the excitation laser beam from the epifluorescence light path. The yellow arrow indicates the laser beam into the fiber cable. Orange arrows indicate the laser beam coupled into the back port of the microscope. (D) The three laser lines: 488 nm, 100 mW CW.; 561 nm, 50 mW CW; and 638 nm, 40 mW laser diode. (E) Single-mode, polarization-maintaining fiber optic patch cable that couples the lasers into the scanning unit. The red box indicates the position of the fiber cable input. (F) Emission filter in front of the APD detector.
• Direct the excitation laser beam into the back port of the microscope. Illuminate the sample dish in epifluorescence mode to visualize transfected cells prior to FFS imaging (Figure 3B).

***Note:*** The microscope was designed to have multiple optical paths: the top-arm transmittance light port and the double-deck turrets, which allow for either epi-illumination or a confocal setup, enabled by two separate excitation light paths generated by a 30/70 beam splitter (Figure 3C). With the APD shutter closed, first use the transmittance light to adjust the objective along the z-axis until the focus is on the top surface of the substrate. Switch to fluorescence mode. Locate cells either through the eyepieces or directly via live-cell imaging.

• The microscope system includes three laser lines: 488 nm (continuous-wave or CW, average power 100 mW, 561 nm (CW, 50 mW), and 638 nm (CW, 40 mW) laser diode in a mounting system with temperature and drive current control, with temperature maintained at 25 °C (Figure 3D).
• Couple the lasers into a single-mode, polarization-maintaining fiber optic patch cable using an achromatic fiber port installed in a fiber port adapter. The light was delivered via fiber to the M612 scanning unit from ISS Inc. (Figure 3E).
• The M612 delivers the excitation light through the right port of the microscope using a dichroic beam splitter designed for the laser excitation wavelength.
• The fluorescence was collected along the same path and focused onto an APD.
• Place the emission filters in front of the APD detector depending on the laser used: 525/50 nm bandpass for 488 nm, 570 nm long pass for 561 nm, or 640 nm long pass for 638 nm (Figure 3F).
• The APD was operated in photon counting mode, with counts sampled by a USB-connected data acquisition card. Photon counts were stored in time-tagged mode, allowing them to be binned at lower frequencies during data analysis.

***Note:*** The maximum sampling frequency used was 2 MHz.

## Step-by-step method details

### Microscope system calibration

**Timing: 4 h**

This section describes the microscope parameter calibration, including measuring the APD detector dead time and the observation volume.

***Note:*** The detector dead time is used during PCH data fitting and only needs to be obtained once per detector. The observation volume is used to calculate protein concentration from the particle number value obtained by PCH analysis. The observation volume describes the PSF of an optical path, and it depends on the numerical aperture (NA) of the objective lens, the pinhole size, and the wavelength of the laser beam. Therefore, the observation volume needs to be measured when any of the above settings change, but not necessarily before every imaging experiment.

1. Measure the APD dead time.
  ***Note:*** The following equation describes the relationship between the observed fluorescence intensity, variance, and detector parameters for a constant light source.^15^
  (Equation 1) $$\begin{array}{c} Q={2p}_{a}-2\left\langle k\right\rangle f_{s}\tau_{d}={2p}_{a}-2I\tau_{d}\; \end{array}$$
  where *p*_*a*_ is the afterpulsing probability, ⟨*k*⟩ is the average photon count rate in counts per sample period, *f*_*s*_ is the sampling frequency in Hz, *τ*_*d*_ is the detector dead time in seconds, and *I* is the fluorescence intensity in counts per second, equivalent to ⟨*k*⟩*f*_*s*_. Q is Mandel’s Q parameter, which is calculated by the following equation:
  (Equation 2) $$\begin{array}{c} Q=\frac{\left\langle {\Delta k}^{2}\right\rangle -\left\langle k\right\rangle }{\left\langle k\right\rangle }\; \end{array}$$
  where ⟨*k*⟩ and ⟨Δ*k*^2^⟩ are the average and variance of the fluorescence intensity.
  a. Prepare 20 μM Atto565 dye solution in water.
    ***Note:*** The concentration must be high enough that intensity fluctuations due to molecular diffusion are negligible.
  b. Apply 400 μL Atto565 dye solution to the center of a glass-bottom dish.
  c. Acquire the fluorescence intensity trace from a single-point FFS measurement for the Atto565 solution using a 561 nm laser beam.
    ***Note:*** See the FFS data acquisition section for the detailed procedures.
  d. Repeat the measurement at different power levels of the excitation laser to acquire different fluorescence intensities.
  e. Calculate Q directly from the fluorescence intensity traces and fit the data to a linear model *Q*= 2*p*_*a*_ - 2*Iτ*_*d*_.
    ***Note:*** Afterpulsing has little effect on PCH fitting so we set *p*_*a*_ = 0.^15^ The dead time is 15 ns for our photon detector.
2. Measure the FFS observation volume.
  To calculate the molecular concentration from the number of particles (N) generated by PCH fitting, we apply the following equation:
  (Equation 3) $$\begin{array}{c} V=\frac{N}{C\times N_{A}}\; \end{array}$$
  where *V* (in liters) is the observation volume of the microscope at a fixed pinhole size, *N* is the number of particles given by PCH fitting, *C* (in molar) is the known molecular concentration of a dye solution, and *N*_*A*_ is Avogadro’s constant. To calculate the observation volume, we independently measured the number of particles (*N*) in a dye solution of known concentration at a fixed pinhole size.
  a. Prepare a 20 nM solution of Atto655 (free acid) in Milli-Q purified water with 0.05% Tween-20 (v/v) in a glass-bottom dish.
    ***Note:*** The concentration should be determined by a spectrophotometer using the published extinction coefficient (ϵ=125,000 M^-1^ cm^-1^).
    ***Note:*** The detergent minimizes the adsorption of dye molecules to the coverslip surface.
  b. Set the pinhole size to be approximately 1 Airy unit (56 μm for our system with a total 80× magnification).^16^
  c. Measure the power of the excitation laser at the microscope turret using a power meter probe. We set the laser power to be 2 μW/cm^2^.
  d. Collect single-point FFS data for 30 s using a 638 nm laser beam.
  e. Obtain the number of particles from the PCH fit by binning the FFS data at a 20 kHz binning frequency and fixing the detector dead time to 15 ns (obtained from the last section).
  f. Calculate the observation volume using the equation above.
    ***Note:*** At 1 Airy unit, our microscope's observation volume is 1.02 fL.

### Seed cells on the glass-bottom dishes

**Timing: 1 h**

This step describes the procedure to seed HEK293T cells on PLL-coated glass substrate.

***Note:*** The protocol should be applicable to other adherent cell types as long as the coating parameters is optimized to facilitate cell adherence.

3. Collect cells of interest using standard cell culture techniques and count the cells.
4. Seed cells in a tissue-culture-level, sterile, PLL-treated, glass-bottom dish or multi-well plate.

***Note:*** Refer to “Coat glass-bottom dishes with poly-L-lysine (PLL) coating buffer” step in the before you begin section. Cell density should be 50–70% at the time of transfection. Make sure the cells are evenly distributed.

### Chemical transfection with DNA plasmids

**Timing: 5 h**

This step describes the procedure to deliver DNA plasmids into cells via chemical transfection.

5. Make the transfection reagent mixture.
  a. For a 14 mm glass-bottom dish, the total amount of DNA is 500 ng, including 50 ng of DNA of interest and 450 ng of empty-vector DNAplasmid (no transgene) to avoid protein overexpression.
  b. Use a 3:1 (μL:μg) ratio for PEI Max:DNA. This is the optimized ratio for chemical transfection in HEK293T cells.
  c. Mix plasmid of interest, empty-vector plasmid, and PEI Max to serum-free DMEM cell medium.
    ***Note:*** The total volume should be 1*/*10 of the cell culture medium in the dish.
  d. Incubate the transfection mixture at room temperature for 20 min.
6. Add the transfection mixture dropwise to the cell culture dish. Gently shake the dish to ensure the transfection mixture is evenly distributed in the growth medium.
7. Incubate cells for 4 h in an incubator maintained at 37°C with 5% CO_2_.
8. Gently aspirate the transfection medium from the dish and add fresh cell growth medium (10% FBS in DMEM).
9. Incubate the transfected cells in the 37 °C cell incubator with 5% CO_2_ overnight.

### Fluorescence cell staining

**Timing: 6 h (up to overnight)**

This step describes the procedure for staining HaloTag-expressing cells with Janelia Fluor HaloTag Ligands (hereafter referred to as the JF dye).

***Note:*** Cell growth medium (10% FBS in DMEM) and imaging medium (10% FBS in DMEM without phenol red) need to be warmed to 37 °C before use.

10. Dilute one vial of JF dye with 1 mL of cell growth medium to make a 5× stock solution (1 μM). Add the growth medium dropwise and gently invert the vial several times until the powder is fully dissolved.

**CRITICAL:** The 1 nmol JF646 vials are designed for single use only. If reused or stored in the freezer, the labeling efficiency may decrease.

11. Further dilute the 400 μL of JF dye stock solution with 1.6 mL cell growth medium to make 2 mL of 1× working solution (200 nM).
12. Carefully remove cell growth medium from the tissue-culture dish without detaching cells.
13. Add 2 mL 1× JF dye solution to the cells slowly against the tissue-culture wall to avoid detaching cells.
14. Incubate cells with the JF solution in the 37 °C cell incubator for at least 6 h to optimize labeling efficiency. Overnight staining is allowed if a pause point is needed.

**CRITICAL:** The labeling time depends on the JF ligand used, cell type, media type, and incubation conditions, which requires case-specific optimization. In our experience, 6 h incubation of 200 nM JF646 solution achieves optimal staining for HEK293T cells grown at a confluence of 80% in a 14-mm glass-bottom dish.

15. Aspirate JF dye-containing growth medium from the tissue-culture dish.
16. Carefully rinse cells with DPBS buffer twice to remove residual dyes.
17. Add 2 mL imaging medium slowly against the tissue-culture wall to avoid detaching cells.

**CRITICAL:** Transfection introduces stress to cells; therefore, all the steps need to be carried out carefully and slowly to avoid disturbance to cells.

### FFS data acquisition

**Timing: 4 h**

This step outlines the procedure for acquiring FFS data with the home-built confocal microscope described in materials and equipment.

18. Turn on and stabilize the laser for at least 10 min before data acquisition.

***Note:*** Before turning on the laser, make sure a “laser in use” safety sign is visible to avoid laser hazard.

**Figure 4:**
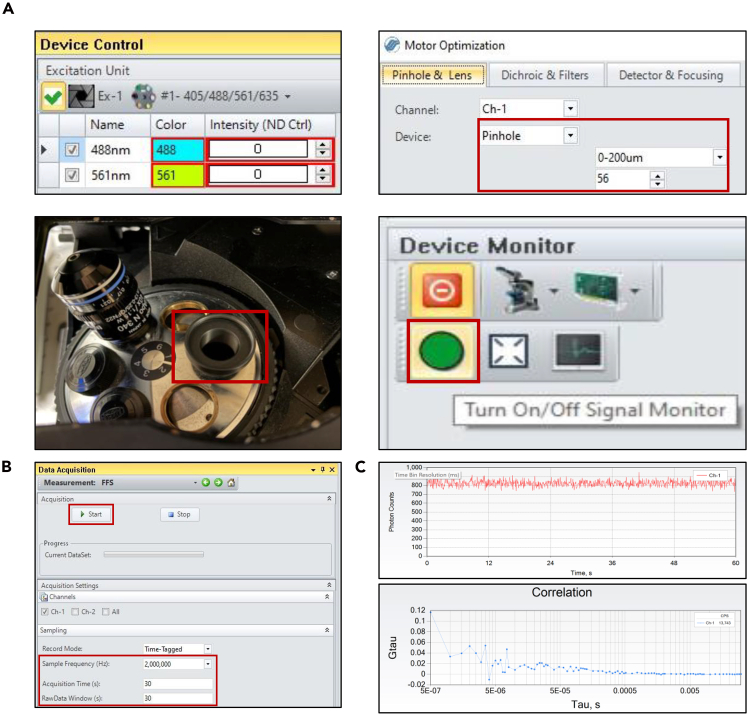
The workflow for FFS data acquisition with a user-interface panel and representative data and analysis (A) Top left: Device Control panel for laser beam selection and intensity adjustment; top right: Motor Optimization panel for adjustment of the pinhole size; bottom left: Turret of the microscope objective for laser power measurement; bottom right: Photon signal monitoring icon. (B) FFS data acquisition panel with adjustable sampling frequency and acquisition time. (C) Top: Raw data for the intensity trajectory; bottom: Autocorrelation plot from FCS analysis.
  b. Adjust the pinhole size to 1 Airy unit (56 μm for our system) (Figure 4A, top right).
22. Measure the laser power at the back aperture of the objective (Figure 4A, bottom left).
23. Add the appropriate immersion solution to the objective before loading the sample.

***Note:*** Water- and oil-immersion lenses are both capable of producing high-quality PCH data. Our laboratory uses a 40× 1.2 NA water-immersion lens to minimize distortions in the point spread function (PSF) across axial depth, since we measure PCH within the cell cytosol. Oil immersion lenses perform better near the coverslip surface/cell membrane interface.^17^ We recommend using oil-immersion lenses only when measurements are performed near the membrane surface, and the microscope is equipped with a piezo-driven objective or stage to precisely control the axial position of the observation volume.

19. Prepare laser power calibration dye solution. For the 638 nm excitation wavelength, we use a 20 nM Atto655 dye solution.
20. Turn on the microscope.
21. Turn on the ISS VistaVision Suite software.
  a. If multiple detectors are involved, make sure the one corresponding to the wavelength of the laser beam is selected (Figure 4A, top left).

24. Bring the objective to the lowest position in the z-axis.
25. Load the plate containing the calibration solution on the microscope.
26. Turn on the photon detector (Figure 4A, bottom right).
27. Slowly raise the objective so that the focus is within the dye solution, where a significant increase in photon counts can be detected.
28. Collect three replicates of FFS data, each lasting for 30 s (Figure 4B).
  a. The photon counts signal should be relatively flat (Figure 4C, top).
  b. The autocorrelation curve should approach zero as the time interval *τ* approaches 1 s (Figure 4C, bottom).
29. Analyze the data following the steps in the PCH data analysis section to acquire N (number of particles) and *ϵ* (brightness). The product of N and *ϵ* indicates the total number of photon counts per second, which should be consistent when measuring the same fluorophores at constant concentration and laser power. This product serves as the reference point to calibrate laser power before starting data acquisition on cell samples. Adjust the laser power and acquire the data until the total photon count rate is consistent with the previous round.

***Note:*** In our system, we used a 638 nm laser beam with a power of 2 μW/cm^2^ measured at the back aperture of the objective. A 20 nM Atto 655 dye solution gives a total photon count rate of 12,000 counts per second (N × *ϵ*).

30. Remove the calibration dye solution and place the cell sample on the stage above the objective. Make sure there’s enough immersion solution between the objective and the cell sample dish.
31. Locate a single cell using either eyepieces or live imaging. Direct the laser focus to the desired imaging spot. Set the desired time and start data collection.

### PCH data analysis

**Timing: 1 h**

This step describes the procedure for obtaining brightness (*ϵ*) and the number of molecules (N) within the focal volume using the Uniform 1 Species model PCH analysis.^18^

**Figure 5:**
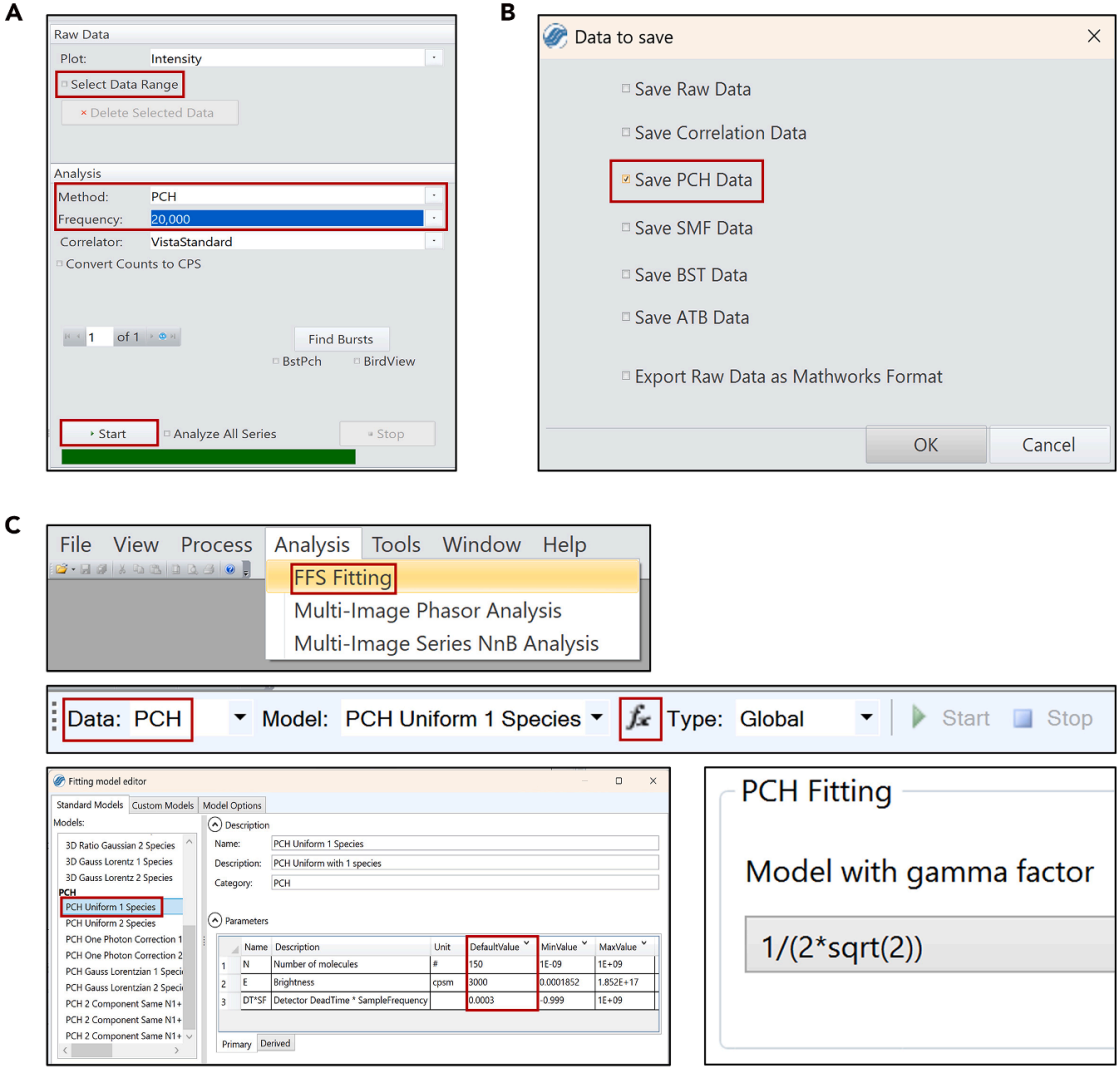
The workflow for PCH analysis (A) PCH analysis panel with options to select data range and sampling frequency. (B) Pop-up window for saving the PCH file. (C) Fitting model editor panel for adjustment of PCH fitting model parameters.
34. Save the PCH analysis by only checking the “Save PCH Data” on the pop-up window (Figure 5B).
35. Open the “FFS Fitting” window under the “Analysis” tab and set the data type to “PCH” and select the “PCH Uniform 1 Species” model.

***Note:*** Different fitting models and a fitting model editor (click the “fx” icon) are available to select the best model and optimize parameters, such as the gamma factor (Figure 5C).

32. Start the ISS VistaVision Suite software and open the FFS file of the acquired data.
33. Perform the PCH analysis on the opened FFS file (Figure 5A).
  a. Select “PCH” for the method under the Analysis tab on the right.
  b. Set the binning frequency as 20 kHz.
  c. Check the photon count intensity across the whole-time frame. A specific time range can be selected for PCH analysis using the “Select data range” option in the top-right.
  d. Once everything is set up, click “Start” to obtain the photon counting histogram.

36. Add all the PCH data files for analysis.
37. Fit the PCH curves.
  a. Set the parameter correction^15^ DT∗SF by multiplying the detector dead time (refer to microscope system calibration) and the sampling frequency. Fix this parameter for all curves.
  b. Set initial parameter estimates for the number of molecules (N) and brightness (*ϵ*).
    ***Note:*** The shape of the PCH contains photon fluctuation resulting from shot noise, diffusion of molecules in an inhomogeneous excitation profile, and particle number fluctuation within the detection volume. These super-Poissonian statistics contain information on both molecular brightness and concentration. The brightness can be calculated using the following equation:
    (Equation 4) $$\begin{array}{c} \gamma \times \varepsilon =\frac{\left\langle {\Delta k}^{2}\right\rangle -\left\langle k\right\rangle }{\left\langle k\right\rangle }\; \end{array}$$
    where *γ* represents the geometry correction factor, which corrects for the excitation volume, ⟨Δ*k*^2^⟩ represents the variance, and ⟨*k*⟩ represents the mean.
    (Equation 5) $$\begin{array}{c} \left\langle k\right\rangle =N\times \varepsilon \; \end{array}$$
    ***Note:*** We set *N* to 150 and *ε* to 3000 cpsm as the initial parameter estimates for our system. The values should be adjusted according to the experimental conditions. *N* will depend on concentration and observation volume size, while *ε* will depend on laser wavelength, laser power, and fluorophore characteristics such as extinction coefficient and quantum yield.
  c. Click the “Start” button at the top to start the PCH fitting process.
38. Assess the fitting quality.
  a. If the fitting process takes too long, re-enter the values for N and ε and refit the data.
  b. In general, a chi-square value around 1.00 is expected from solution/buffer measurements, while values between 1.00 and 4.00 are often observed from live cell measurements.^19^

### HaloTag dimer-monomer brightness ratio calibration

**Timing: 3 days**

This step describes the calibration of the HaloTag dimer-to-monomer brightness ratio to calculate labeling efficiency.

***Note:*** Although pre-synthesized small-molecule fluorophores do not require fluorophore maturation as typical genetically coded fluorescent proteins do, the intracellular diffusion barrier, misfolded self-labeling proteins, and steric hindrance resulting from linker constraints or structural crowding (the covalent attachment of the first bulky dye blocking or distorting the entry tunnel of the adjacent HaloTag domain) could cause incomplete labeling. Therefore, in this section we elaborate on the steps to measure and calculate labeling efficiency *p*_*f*_. This value is used for calculating the relative brightness in the “Normalization and correction of brightness values”.

39. Seed HEK293T cells (refer to seed cells step) on two PLL-coated glass-bottom dishes (refer to coat dishes step).
40. Transfect the cells at 70% confluence with 1×HaloTag in one dish and with 2×HaloTag in the other (refer to chemical transfection step).
41. Stain the cells with JF646 HaloTag dye solution for at least 6 h (refer to stain cells step).
42. Wash the cells with PBS buffer three times before imaging and incubate cells with the imaging medium.
43. Collect single-point FFS data for 30 s at six different positions for each cell. Acquire data from at least 10 cells for 1×HaloTag- and 2×HaloTag-transfected cells (refer to FFS data acquisition step).
44. Perform PCH analysis on the FFS data using a 20 kHz sampling frequency (refer to PCH data analysis step).
45. Fit the PCH data with the “PCH Uniform 1 Species” model to calculate N and *ε* values for each position for individual cells.
46. Calculate the average brightness values for individual cells expressing 1×HaloTag cells or 2×HaloTag by averaging the brightness values collected from six different positions.
47. Calculate the dimer-to-monomer brightness ratio. Subtract 1.0 from this ratio to obtain the labeling efficiency (*p*_*f*_).

***Note:*** We typically obtain a dimer-to-monomer ratio of 1.7, suggesting that the labeling efficiency is 0.7.

### Determination of monomeric protein concentration

**Timing: 10 min**

This step describes the calculation of monomeric protein concentration before the proteins assemble into higher-order oligomeric states.

***Note:*** For proteins that exist as a mixture of different oligomers in the cells, it is important to know their protomer (monomer) concentration. Assuming the JF646 HaloTag ligand brightness does not depend on the protein oligomerization state, the total count rate of the photons should not change before and after oligomerization.

48. Calculate apparent protein concentration (in nanomolar) based on the number of particles (N) using the following equation:

(Equation 6) $$\begin{array}{c} C=\frac{N\times 10^{9}}{V\times N_{A}}\; \end{array}$$

where V is the observation volume in liters (refer to Microscope system calibration), N is the number of particles measured by PCH, and *N*_*A*_ is Avogadro’s constant.

49. To further recover the protomer concentration, we used the following equation:

(Equation 7) $$\begin{array}{c} C_{mono}=\frac{C_{app}\times \varepsilon_{app}}{\varepsilon_{mono}\times p_{f}}\; \end{array}$$

where *ε*_*mono*_ is the HaloTag monomer brightness, and *p*_*f*_ is the fluorescence probability or labeling efficiency.

### Normalization and correction of brightness values

**Timing: 10 min**

This section describes how to normalize and correct molecular brightness measurements to enable comparison across different experimental conditions.

***Note:*** After these corrections, the data are normalized to the monomeric brightness of the fluorophore and corrected for partial labeling, so that a relative brightness value (*ε*_*corrected*_) of 2-fold corresponds to a fully dimeric population.

50. Calculate normalized brightness $\varepsilon_{norm}$ by $\frac{\varepsilon_{app}}{\varepsilon_{mono}}$.
51. Use *ε*_*norm*_ directly as the corrected relative brightness when *ε*_*app*_ ≤ *ε*_*mono*_ (due to experimental uncertainty or noise). When *ε*_*app*_ ≥ *ε*_*mono*_, correct the normalized brightness ratio *ε*_*norm*_ for any inefficient labeling by applying the following equation^9^:

(Equation 8) $$\begin{array}{c} \varepsilon_{corrected\;}=\frac{\varepsilon_{norm}-1}{p_{f}}+1\; \end{array}$$

52. Use the *ε*_*corrected*_ value to compare the oligomerization state of the same HaloTag fusion protein with the same staining ligand under different physiological conditions in living cells with temporal resolution.^1^

## Expected outcomes

In a single imaging session, this protocol enables the acquisition of FFS data and the extraction of intracellular protein concentration and assembly-state information through PCH analysis. In this section, we use a photoactivatable protein, the *Arabidopsis thaliana* cryptochrome 2 photolyase homology region fused to an N-terminal HaloTag (HaloTag-AtCRY2PHR), to demonstrate how to: 1) determine intracellular protein concentration and oligomerization state before and after blue-light stimulation, and 2) quantify the dark-state dissociation of AtCRY2PHR oligomers.^20^

**Figure 6:**
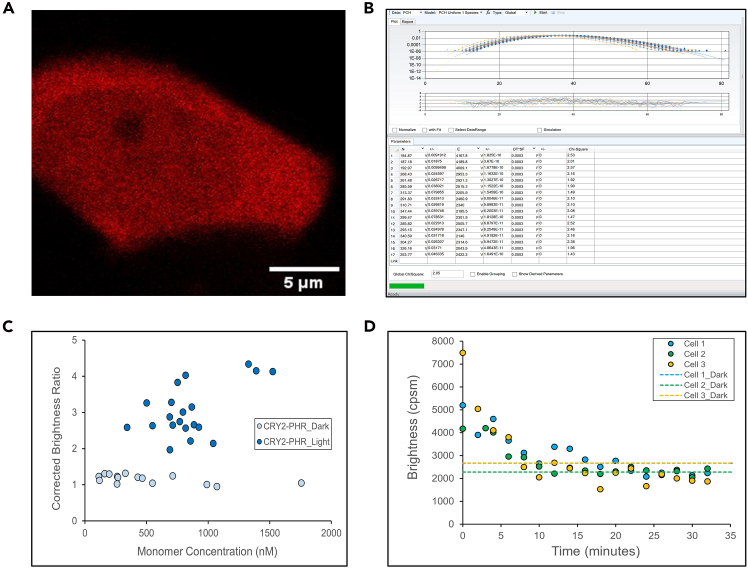
The workflow of extracting molecular brightness in HEK293T cells with PCH analysis (A) Confocal image of a single HEK293T cell expressing HaloTag-AtCRY2PHR stained with JF646-conjugated HaloTag ligand. (B) PCH data fitting using the Uniform 1 Species model. (C) Comparison of oligomerization states of AtCRY2PHR protein vs. concentration before and after blue light stimulation. Measurements were not paired; for cells stimulated by light, the monomer-equivalent concentration was back-calculated (see the main text). (D) Dark recovery analysis of the AtCRY2PHR protein in three individual living HEK293T cells. After 5 min of blue light stimulation on the cells, time-stamped intensity trajectories were collected every 2 min, each lasting for 30 s, at the same spot.

HEK293T cells were transfected with HaloTag-AtCRY2PHR and labeled with JF646 dye for 6 h prior to imaging. To establish base-level oligomerization, transfected cells were identified using JF546 fluorescence under 561 nm laser excitation (Figure 6A). Six cytoplasmic positions per cell were selected for FFS acquisition (30 s per position; 561 nm excitation, 2 μW/cm^2^ measured at the back aperture of the objective). Approximately 20 cells were analyzed to determine the basal oligomerization state of AtCRY2PHR, which was primarily monomeric. To induce oligomerization, the same batch of cells was exposed to continuous blue-light for 5 min using a 470 nm LED light source (6 mW/cm^2^ measured at the sample plane), followed immediately by single-point FFS data acquisition for 30 s. To determine oligomer dissociation kinetics following blue light withdrawal, cells were first stimulated with pulsatile 470 nm illustration (6 mW/cm^2^, 5 min), followed by time-resolved FFS acquisition over 30 min. FFS measurements were carried out every 2 min using a single observation spot, consisting of 30 s data acquisition followed by 90 s dark incubation. Control experiments confirmed that 561 nm excitation alone does not induce AtCRY2PHR oligomerization. PCH analysis was subsequently performed on the FFS datasets using a 20 kHz binning frequency. Brightness (*ε*) and particle number (N) values were obtained by fitting the PCH curves using the “PCH Uniform 1 Species” model. Prior to fitting, the DT∗SF parameter (dead time∗sampling frequency) was set to 0.0003 and held constant for all datasets. Following fitting, each PCH file generated values for brightness (*ε*) and the number of particles within the observation volume (N) (Figure 6B). Fit quality was evaluated using the chi-square statistics, where values below 2 indicated a good agreement between the experimental data and the fitting model.

To examine the concentration-dependent assembly behavior, average brightness values were plotted against the average recovered monomer concentration, using data collected from six positions within each cell (Figure 6C). The relative brightness value was used as a proxy for oligomeric state. We observed that CRY2PHR undergoes light-dependent oligomerization and that the assembly state of the oligomer mixture increases with protein concentration. Specifically, CRY2PHR reached a brightness value of approximately 4 at intracellular concentration above 1 μM, consistent with predominate tetramer formation. At concentrations below 1 μM, relative brightness values ranged from 1 to 4, indicating heterogeneous oligomeric populations. Assuming a simplified two-state equilibrium model consisting of monomers and tetramers, the fraction of oligomerized protein can be estimated for individual cells. However, immediate oligomeric species, such as dimers, are likely present in the cell. Therefore, direct resolution of oligomer distributions may require complementary approaches, such as super-resolution imaging or electron microscopy. Following pulsatile 470 nm excitation, CRY2PHR oligomers gradually dissociated back towards the monomeric state, with recovery occurring within approximately 20 min after blue-light withdrawal (Figure 6D).

## Limitations

PCH and fluorescence correlation spectroscopy (FCS) both belong to FFS.^7^ Whereas FCS measures the decay of the intensity fluctuation correlation *G*(*τ*) to determine the characteristic timescale over which the molecule remains within the detection volume, PCH measures the amplitude of intensity fluctuations. Although FCS can imply concentration from the amplitude of ⟨*G*(0)⟩, PCH is more sensitive to molecular brightness. However, the PCH curve approaches a Poisson distribution as the fluorophore concentration increases because fluctuations in the number of fluorophores in the detection volume and diffusion-induced intensity fluctuations both approach zero.

This scenario sets a practical upper limit on PCH’s concentration estimation. To maximize the difference between PCH and a Poisson distribution, one can either reduce the fluorophore concentration or increase molecular brightness. Considering the photon count detection limit (2 million photons/s) from APD, in our experience, a decent PCH fit can be achieved at up to 1.2 μM of HaloTag-fusion protein. In our hands, the UBC promoter, which results in lower expression than other high-copy promoters (e.g., CMV), works well in HEK293T cells with transient transfection. Lentiviral transduction is expected to yield better results due to its more homogeneous expression profile and titer-dependent expression.^21^

PCH fails when the sampling time is too long relative to the molecular diffusion time, because the intensity fluctuations are averaged out, leading to errors in determining molecular brightness. In our case, we used a 2 MHz sampling frequency (0.5 μs sampling time) to ensure sufficient sampling events of the target proteins within the detection volume. Based on the Einstein-Stokes equation, assuming the diffusion coefficient of a protein molecule is 10^*−*6^-10^*−*7^cm^2^/s, the average residence time in a diffraction-limited focal spot (300 nm) is about 0.2–2 ms, much longer than the sampling time. The other extreme case is that, if diffusion is *so* slow that the particles are essentially frozen in the detection volume, the fluctuations needed for PCH analysis may not occur over the measurement time. However, in most biological contexts, slow diffusion should still allow PCH to work.

The single-point measurement loses direct access to the spatial distribution of fluorescence fluctuations. In other words, PCH is not a microscopy technique. Alternative approaches, e.g., Number and Brightness analysis, should be employed if the spatial distribution of protein oligomerization is needed. However, one needs to ensure that the sampling time (which depends on the camera’s frame rate) is shorter than the characteristic timescale of fluctuations (e.g., diffusion) to capture intensity fluctuations accurately.

Finally, this protocol measures an average brightness using the one-species fitting procedure. Using a monomer-oligomer two-state model, the percentage of each state can be derived. However, if there are more than two oligomeric components, it would be extremely difficult to figure out the weight of each species by PCH alone. Complementary strategies, e.g., electron microscopy or super-resolution imaging, should provide greater insight into the composition of the oligomer mixture.

## Troubleshooting

### Problem 1

Transfection and staining can cause significant stress in cells if not performed properly, resulting in irregular cell morphology, impaired nuclei, and molecular aggregates. This could occur during the chemical transfection step when changing the cell medium after a 4 h incubation.

### Potential solution

Use low-passage cells and ensure no mycoplasma contamination. Check that cell morphology is normal before conducting the imaging experiment.

### Problem 2

Slow intensity fluctuations (on the order of seconds to minutes) may arise from cell movement or from large oligomeric species diffusing in and out of the observation volume, leading to large chi-square values after PCH fitting and analysis. This could occur during the PCH data analysis step.

### Potential solution

Check the spot for FFS data collection and ensure it is within a homogeneous cellular compartment. Avoid collecting FFS data near membrane boundaries or subcellular compartments with inhomogeneous fluorescence distribution. During the PCH fitting, the data range can be manually selected to exclude any unusual fluctuation. However, we recommend a minimum of 15 s to generate high-quality PCH data.

### Problem 3

The fluorescence intensity is too high for FFS data acquisition due to the high expression level of the HaloTag fusion protein. This could occur during the FFS data acquisition step.

### Potential solution

Switch to a lower-expressing promoter for the plasmid. However, expression levels naturally vary due to cell-to-cell variation. Avoid selecting very bright cells in a field of view. Use less recovery time after transfection before staining.

### Problem 4

Measured molecular brightness values are lower than the expected monomer brightness, preventing reliable resolution of oligomeric states. This could occur during the PCH data analysis step.

### Potential solution

Molecular brightness values depend strongly on excitation power, fluorophore properties, optical alignment, detector settings, and fitting parameters. Therefore, absolute brightness thresholds are instrument-dependent and should always be interpreted relative to the experimentally determined monomer brightness under identical imaging conditions. First, verify that the fitting software correctly applies the observation-volume shape correction (gamma factor), as the correction differs between one-photon and two-photon excitation conditions.^7^ Incorrect gamma-factor settings can substantially underestimate molecular brightness values. Next, perform a calibration measurement using the same fluorophore or dye in a monomeric state at a known low concentration (e.g., 10–20 nM) under the identical optical configuration and excitation power used for cellular measurements. The measured monomer brightness should establish the reference value for subsequent oligomerization analysis. If cellular measurements produce brightness values lower than the calibrated monomer reference, possible causes include insufficient excitation power, suboptimal optical alignment, detector nonlinearity, incomplete fluorophore labeling, or excessively high intracellular fluorophore concentrations. High fluorophore concentrations can reduce apparent molecular brightness because multiple fluorophores simultaneously occupy the observation volume, thereby compromising accurate PCH fitting and oligomer-state determination.

### Problem 5

Inconsistent laser calibration measurement between individual imaging sessions. This could occur during the FFS data acquisition step.

### Potential solution

Check laser power stability and ensure the objective is focused into a similar z-position each time. A piezo stage helps ensure that the focal plane is consistent across individual experiments.

## Resource availability

### Lead contact

Requests for further information and resources should be directed to and will be fulfilled by the lead contact, Kai Zhang (kaizkaiz@illinois.edu).

### Technical contact

Requests for technical details and should be directed to and will be fulfilled by the technical contact, Zixiao Li (zixiaol2@illinois.edu).

### Materials availability

Plasmids generated in this study are in the process of being deposited with Addgene.

### Data and code availability

Data reported in this paper will be shared by the lead contact upon request. The PCH fitting was performed using the commercial software VistaVision 64 4.2.584, provided by ISS. This paper does not report additional code. Any additional information required to reanalyze the data reported in this paper is available from the lead contact upon request.

## Acknowledgments

This work is supported by the American Parkinson Disease Postdoctoral Fellowship (award no. 978673) (to T.C.); the 10.13039/100000057National Institute of General Medical Sciences (R01GM132438); the 10.13039/100000025National Institute of Mental Health (R01MH124827), 10.13039/100000001NSF (award no. 2121003); and an NSF Science and Technology Center for Quantitative Cell Biology grant (award no. 2243257) (to K.Z.). We are grateful to Dr. Yuansheng Sun from ISS for insightful comments and support in building the microscope and PCH analysis. We thank Dr. Valerica Raicu lab at the University of Wisconsin–Milwaukee for sharing molecular cloning strategies.

## Author contributions

Conceptualization, T.C. and K.Z.; methodology, Z.L., T.C., and K.Z.; investigation, Z.L., T.C., and Y.L.; formal analysis, Z.L. and T.C.; visualization, Z.L., T.C., and K.Z.; data curation, Z.L. and T.C.; writing – original draft, Z.L.; writing – review and editing, Z.L. and K.Z.; funding acquisition, T.C. and K.Z.; resources, K.Z.; supervision, K.Z.

## Declaration of interests

The authors declare no competing interests.
